# Moss-pathogen interactions: a review of the current status and future opportunities

**DOI:** 10.3389/fgene.2025.1539311

**Published:** 2025-02-11

**Authors:** Huan Zhang, Qilin Yang, Leyi Wang, Huawei Liu, Daoyuan Zhang, Cheng-Guo Duan, Xiaoshuang Li

**Affiliations:** ^1^ State Key Laboratory of Desert and Oasis Ecology, Key Laboratory of Ecological Safety and Sustainable Development in Arid Lands, Xinjiang Institute of Ecology and Geography, Chinese Academy of Sciences, Urumqi, China; ^2^ College of Resources and Environment, University of Chinese Academy of Sciences, Beijing, China; ^3^ Xinjiang Key Lab of Conservation and Utilization of Plant Gene Resources, Xinjiang Institute of Ecology and Geography, Chinese Academy of Sciences, Urumqi, China; ^4^ Key Laboratory of Plant Design, National Key Laboratory of Plant Molecular Genetics, Shanghai Center for Plant Stress Biology, CAS Center for Excellence in Molecular Plant Sciences, Chinese Academy of Sciences, Shanghai, China

**Keywords:** moss, pathogen, interaction, disease resistance mechanism, plant immune receptors

## Abstract

In complex and diverse environments, plants face constant challenges from various pathogens, including fungi, bacteria, and viruses, which can severely impact their growth, development, and survival. Mosses, representing early divergent lineages of land plants, lack traditional vascular systems yet demonstrate remarkable adaptability across diverse habitats. While sharing the fundamental innate immune systems common to all land plants, mosses have evolved distinct chemical and physical defense mechanisms. Notably, they exhibit resistance to many pathogens that typically affect vascular plants. Their evolutionary significance, relatively simple morphology, and well-conserved defense mechanisms make mosses excellent model organisms for studying plant-pathogen interactions. This article reviews current research on moss-pathogen interactions, examining host-pathogen specificity, characterizing infection phenotypes and physiological responses, and comparing pathogen susceptibility and defense mechanisms between mosses and angiosperms. Through this analysis, we aim to deepen our understanding of plant immune system evolution and potentially inform innovative approaches to enhancing crop disease resistance.

## 1 Introduction

Mosses are characterized by their diminutive stature and relatively simple organization. Their gametophytes and sporophytes predominantly comprise a single layer of cells and lack true vascular tissue, placing them in the category of non-vascular plants. Bryophytes, which include mosses, liverworts, and hornworts, are important representatives of early terrestrial plants that evolved from aquatic plants around 450 million years ago (Clarke et al., 2011; Delaux et al., 2019; Resemann et al., 2019). These mosses represent an ancient lineage in plant evolution, having diversified over at least 300 million years (Dobbeler, 1997). Mosses hold a foundational position in the evolution of terrestrial plants and play a crucial role in the transition from aquatic plants to terrestrial plants, linking single-celled green algae to vascular plants (De León and Montesano, 2013; De León and Montesano, 2017). Additionally, they serve as an effective model organism (De León, 2011) due to their compact size, simple structure, strong regenerative ability, and short cultivation cycle (Schaefer, 2002; Cove et al., 2006). Furthermore, mosses are valuable for molecular biology and genetic studies because they respond similarly to environmental signals and plant growth factors as other vascular plants, and their life cycle is dominated by the haploid gametophyte stage (Schaefer and Zryd, 1997; Schaefer, 2002).

Mosses, like other plants, can be infected by various pathogens including fungi, bacteria, oomycetes, and viruses. Among these, several key model pathogens have been instrumental in understanding moss-pathogen interactions. *Botrytis cinerea*, one of the most extensively studied fungal pathogens, serves as a primary model system due to its ability to directly penetrate the cell wall and invade intercellular spaces of *Physcomitrella patens*, causing tissue browning and death (Yan et al., 2018). Similarly, the bacterial pathogen *Pseudomonas syringae* pv. *tomato* DC3000 has emerged as another important model organism, as it can effectively colonize *P. patens* and induce characteristic disease symptoms, providing valuable insights into the conservation of immune responses between mosses and vascular plants (Yan et al., 2023). Other significant pathogens include *Pectobacterium carotovorum*, which causes tissue maceration in *P. patens* (Alvarez et al., 2016), and the oomycetes *Pythium irregulare* and *P. debaryanum*, which infect multiple tissue types and cause browning of the stem, midrib, and leaf base (Oliver et al., 2009). While less studied, viral infections have also been documented, with *Tobacco Mosaic Virus* (TMV) and *Cucumber Green Mottle Mosaic Virus* (CGMMV) detected in moss species such as *Polytrichum commune* (Polischuk et al., 2007). Understanding these diverse pathosystems is crucial for elucidating the evolution of plant-pathogen relationships and may provide insights into developing disease resistance strategies in both non-vascular and vascular plants. Due to their significant evolutionary position and relatively simple morphological structure, mosses are regarded as an ideal model system for studying plant-pathogen interactions. Research on mosses allows for a clear understanding of the mechanisms behind plant disease resistance responses, as well as insights into the defense strategies of early land plants and their evolutionary trajectories. This knowledge contributes to better understanding of how pathogens threaten major crops and how plants activate their complex defense mechanism, which could aid in managing or preventing crop diseases. This review aims to provide a comprehensive overview of studies on moss-pathogen interactions. It examines the susceptibility of mosses compared to angiosperms to various pathogens and highlights the similarities and differences in the defense mechanisms activated after infection. Additionally, it discusses the interactions between various hosts and pathogen and the physiological phenotypes in mosses. Investigating these interactions is crucial for identifying key factors of early land plant defense systems and for elucidating the molecular mechanisms of plant-pathogen interactions from an evolutionary perspective. This review will definitely provide valuable insights for molecular breeding of plant disease resistance.

## 2 Mosses-pathogen interactions

### 2.1 Mosses-fungus interactions

Fungal pathogens have been identified in moss populations, and the associated disease symptoms have been documented for decades (Tsuneda et al., 2001a; De León, 2011). Various fungi, including *Thyronectria hyperantartica*, *Teprocybe palustris*, *Bryoscyphus dicrani*, *Scleroconidioma sphagnicola*, *Acrospermum adenum*, *Ardapia retitruga*, *Lizonia baldinii,* and *Atradidymella muscivora,* cause necrotic lesions and the death of moss gametophytes (Davey and Currah, 2006; Davey et al., 2009). Some of these pathogens have been studied for their ability to penetrate bryophyte tissues, destroy cells, and elicit host responses (Martínez-Abaigar et al., 2005; Davey and Currah, 2006; Davey et al., 2009). The invasion of moss pathogens into host cells typically involves vegetative hyphae, osmotic plugs, and sometimes appressoria, along with the enzymatic digestion of plant cell walls (Tsuneda et al., 2001a; Davey and Currah, 2009; Davey et al., 2009). Research has particularly focused on the interaction between *P*. *patens* and *B*. *cinerea*. *B*. *cinerea* can directly penetrate the cell wall of *P*. *patens* and invade the intercellular space, which can cause brown spots in moss leaves. With the extension of time, the number of brown spots increases and expands, spreading from the base and tip of the leaves to the middle, eventually leading to leaf decay and plant death, showing wilting symptoms (Yan et al., 2018). A strain isolated from *Leucobryum glaucum*, when introduced to *P. patens*, produced symptoms similar to *L. glaucum* blight disease. This fungus was closely related to *Mucor racemosus* based on sequencing, indicating that *M. racemosus* can infect both *P. patens* and *L. glaucum*, causing wilting, chlorosis, and other symptoms, although the detailed infection mechanisms are not fully understood (Liu, 2011). In contrast, *M. racemosus* does not infect *Haplocladium microphyllum* or *Mnium hornum* (Liu, 2011). *Colletotrichum gloeosporioides* can infect *P. patens* by directly penetrating the host cell wall, primarily damaging the leaves and resulting in tissue maceration, necrosis, etc., (Reboledo et al., 2015; Otero-Blanca et al., 2021). Additionally, *Verticillium dahliae* can also infect *P. patens*, *Bryum argenteum*, and *Syntrichia caninervis*, causing tissue browning and chloroplast degradation, with leaves and stems being the primary sites of infection. Infected mosses exhibit localized lesions and browning at the leaf edges. *Sphagnum fuscum* can be infected by *Lyophyllum palustre*, *S. sphagnicola, Oidiodendron maius, Acremonium cf. curvulum, Arrhenia retiruga* and *Pochonia bulbillosa*, where the hyphae selectively degrade the moss cell wall, leading to wavy deformations as they grow both outside and inside the cell wall, resulting in wavy deformation of the cell wall (Untiedt and Muller, 1985; Tsuneda et al., 2001b; Davey and Currah, 2006). *Rozellopsidalean fungi* can penetrate protonema and rhizoid cells to infect the *Funaria hygrometrica*, *Bryum pseudotriquetrum* and *Bryum capillare* (Martínez-Abaigar et al., 2005), resulting in compression of moss cell tissues, the release of numerous lipid droplets, necrosis of cortex cells in stems and leaves, and chloroplast detachment. *Nectri mnii* has also been observed to spread throughout the stem tissues of *Plagiomnium medium* (Döbbeler, 1988; Lawton and Saidasan, 2009). In contrast, the interaction between mosses and fungi has not been thoroughly examined. For instance, *Phyllosticta tetraplodontis* Lebedeva causes browning and chlorosis of the sporophytic tissues of *Tetraplodon*, ultimately resulting in the loss of the capsules. The process of cell disruption and degradation for this organism remains unidentified (Davey and Currah, 2006). Detailed descriptions of fungal infections in mosses were listed in Table 1.

**TABLE 1:** Interaction between mosses and fungal pathogens.

Mosses species	Pathogens	Intrusive behavior	Ultrastructural changes	Reference
*Physcomitrium patens*	*Botrytis cinerea*	The formation of penetrating nails directly penetrates the cell wall and invades the intercellular space	The cell wall undergoes localized changes, including the formation of papillary structures, and the destruction of organelle structures	Yan et al. (2018)
*Physcomitrium patens*	*Mucor racemosus*	--	Protoplasts shrink to collapse, resulting in a decrease in the number of chloroplasts	Liu, (2011)
*Physcomitrium patens*	*Colletotrichum gloeosporioides*	Directly penetrate the host cell wall	Chloroplast repositioning	Otero-Blanca et al. (2021)
*Physcomitrium patens*	*Verticillium dahliae*	Penetrate the cell wall and continue to extend	Cell wall browning and chloroplast degradation	Lawton and Saidasan, (2009)
*Funaria hygrometrica*	*Rozellopsidalean fungi*	The nail-like structure penetrates proton cells and root cells	Produced deeply brown-pigmented ingrowths of wall material surrounding sites of fungal penetration	Martínez-Abaigar et al. (2005)
*Bryum pseudotriquetrum*	*Rozellopsidalean fungi*	Form pink or light brown round to pear-shaped tip cells in the primary branch	Cell tissue compression releases a large amount of lipid droplets	Martínez-Abaigar et al. (2005)
*Bryum capillare*	*Rozellopsidalean fungi*	Penetrating apical cells and apical cells of primary lateral branches, the rhizoids are large	Loss of cytoplasmic contents	Martínez-Abaigar et al. (2005)
*Hylocomium splendens*	*Atradidymella muscivora*	Mycelia or attachment cells penetrate the cell wall	Dark sediment and lack of chloroplasts in the invaded area	Davey et al. (2009)
*Aulacomnium palustre*	*Atradidymella muscivora*	Mycelia or attachment cells penetrate the cell wall	Dark sediment and lack of chloroplasts in the invaded area	Davey et al. (2009)
*Polytrichum juniperinum*	*Atradidymella muscivora*	Mycelia or attachment cells penetrate the cell wall	Dark sediment and lack of chloroplasts in the invaded area	Davey et al. (2009)
*Funaria hygrometrica*	*Atradidymella muscivora*	Nutrient hyphae penetrate the cell wall or the compressed material with a dome shaped swelling to produce penetrating nails	The surface of the gametophyte produces flocculent material, white aerial hyphae, and cell wall degradation	Davey et al. (2009)
*Sphagnum fuscum*	*Scleroconidioma sphagnicola*	Penetrate the cell wall	Necrosis of cortical cells and detachment of chloroplasts in stems	Tsuneda et al. (2001b)
*Spagnum fallax*	*Tephrocybe palustris*	Forms penetration pegs that locally produce pectinases to digest the middle lamella between leaf cells	Cause host protoplast degeneration	Redhead and Spicer (1981), Untiedt and Muller (1985), During and Vantooren (1990)
*Plagiomnium medium*	*Nectria mnii*	The cell penetrating nail penetrates the host cell, and the cell wall digestion only occurs at the advancing tip of the nail	Replacing host cell protoplasts with intracellular hyphae	Döbbeler, (1988)
*Sphagnum*	*Bryophytomyces sphagni*	Fungal propagules replace the moss spores	--	Chau (1979), Redhead and Spicer (1981)
*Sphagnum squarrosum*	*Discinella schimperi*	Forming appressorium highly branched caps on apical mucilage cells on stem apex	--	Prior (1966); Redhead and Spicer (1981)
*Sphagnum fuscum*	*Oidiodendron maius*	Decomposing moss cell walls	Creating localized voids in cell wall	Tsuneda et al. (2001a), Rice et al. (2006)
*Sphagnum fuscum*	*Acremonium cf. curvulum*	Degrading the leaf cell wall	Appear tortuous microfiber elements, and the wall layer gradually form local voids	Tsuneda et al. (2001a)
*Sphagnum fuscum*	*Arrhenia retiruga*	Invades the host cell channel	--	Davey and Currah, (2006)
*Tetraplodon*	*Phyllosticta tetraplodontis*	--	--	Davey and Currah, (2006)
*Orthotrichum diaphanum*	*Octospora orthotricha*	--	Formation of a gall on the rhizoid	Davey and Currah, (2006)
*Sphagnum fuscum*	*Pochonia bulbillosa*	Selective degradation of cell wall	Hyphae grows both outside and within cell wall, creating wavy deformations of the wall	Davey and Currah, (2006)

### 2.2 Mosses-oomycete interactions

There have been few studies on the interaction between mosses and oomycetes. Two oomycetes, *P*. *irregulare* and *P*. *debaryanum*, can develop appressoria to penetrate moss tissues, affecting all tissue types including leaves, protonema, rhizoids and stems. This leads to the browning of the stem, midrib and leaf base in *P. patens* Appressoria are visible during the early stages of infection. When several cells are infected, multi-digital haustoria-like structures appear in moss-infected tissues. The penetration of host cell walls into adjacent cells occurs through constricted hyphae. As the duration of infection increases, hyphal colonization becomes more extensive, leading to tissue rot in *P. patens*, shrinkage of cytoplasm, relocation of chloroplasts, and browning (Oliver et al., 2009). The oomycete *Pythium ultimum* cause the formation of areas of dying and dead moss gametophytes, while symptoms such as chlorosis and necrosis, followed by the death of gametophyte, are typical of all known mosses pathogens (fungi, oomycetes, and bacteria). (Redhead and Spicer, 1981; Untiedt and Muller, 1985; Döbbeler, 2003; Davey and Currah, 2006; De León, 2011), The mechanisms of penetration and destruction of moss cells by different pathogens, the causes of disease, and the host‘s response to infection are different (Hühns et al., 2003; Oliver et al., 2009; Alvarez et al., 2016).

### 2.3 Mosses-bacteria interactions

There are currently few reports on the interactions between moss and bacteria. *Erwinia carotovora* can infect *P. patens* (De León et al., 2007) by entering through wounds, resulting in damage in moss tissue, browning of gametophytes, and gradual rotting. This bacterium can also cause cytoplasmic shrinkage, accumulation of autofluorescent substances, changes in chloroplast structure, and pigments in *P. patens*. *P*. *carotovorum* produces a high concentrations of enzymes that break down plant cell walls, including cellulases, proteases and pectinases (Toth and Birch, 2005). These enzymes work synergistically with other virulence factors to dissolve host tissues and facilitate host cell death (Davidsson et al., 2013). *P. patens* is susceptible to *P. carotovorum* infection (Alvarez et al., 2016), and after infection, *P. patens* exhibits basal tissue browning and partial wilting within 24 h (Alvarez et al., 2016). The proteases and cell wall-degrading enzymes degrade moss tissues leading to cell death. Furthermore, *P. syringae* is recognized as one of the most destructive agricultural pathogens (Liu et al., 2020). *P. syringae* pv. *tomato* DC3000 (*Pst* DC3000) can also infect *P. patens*, *B. argenteum* and *S. caninervis*, causing typical disease symptoms such as wilting and browning of the stems and leaves, etc., (Yan et al., 2023).

### 2.4 Mosses-virus interactions

Viruses represent a major class of pathogens on earth that utilize their own viral suppressors of RNA silencing (VSRs) (Duan et al., 2012; Qiao et al., 2013; Yin et al., 2019) to overcome plant defenses and infect all types of organisms (Retel et al., 2019). Although the small RNA pathways in mosses and vascular plants share high similarity, little is known about the infection of mosses with viral pathogens (Marttinen et al., 2022). Researchers detected infections by TMV and CGMMV in Antarctic *P*. *commune*, as well as TMV infection in *Barbilophozia attenuate* (Polischuk et al., 2007). These two viruses typically infect dicotyledonous plants, and it remains unclear how they naturally infect mosses cells in the field. Additionally, viruses have been detected in the phyllosphere of *Sphagnum*, which serves as an important source of virus diversity and activity (Marttinen et al., 2022). Furthermore, it has been demonstrated that *P. patens* is infected with Tomato Bushy Stunt Virus (TBSV) and Cucumber Mosaic Virus (CMV) (Marttinen et al., 2022), which primarily damaging its gametophyte. This could facilitate the investigation of virus-moss host interactions using *P. patens* as a laboratory viral host.

In summary, mosses can interact with pathogens, particularly fungi, throughout their life cycles. The infection sites are varied, and there is no clear preference for specific tissues. *P. patens* is the main moss model used in current research, with a notable focus on the *Physcomitrium-*moss relationship. However, studies on other moss species are still quite limited. It is necessary to strength the research that elucidate the mechanisms behind bacterial and viral infections, as these pose major challenges in comprehending moss disease resistance.

## 3 Comparison of mosses and vascular plant susceptibility to pathogens

We compared the symptoms and incidence rates of representative moss *P. patens* against the vascular plants *Arabidopsis thaliana* or tobacco after pathogen infection. It discovered that in most cases, the disease symptoms in infected mosses were similar to those in vascular plants, but the disease development rate was often faster than that in vascular plants (Table 2). Lesions can be seen on the tissue surface within 24 h of *B. cinerea* infecting *P. patens* and the moss dies after 5 days infection. Similarly, when *B. cinerea* infects *A*. *thaliana,* after 1-day, necrotic lesions appeared on the leaves. The symptoms are similar in the two hosts, mainly manifesting as lesions on leaves, wilting and eventual death (González et al., 2006; Jiang et al., 2013; Yan et al., 2018). For *B*. *cinerea*, the onset time of the two plants is not much different. The leaves of *P*. *patens* dipped with spore suspension of *V*. *dahliae* showed typical V-shaped necrotic spots within 15 days, while the leaves of *A*. *thaliana* dipped with spore suspension of *V*. *dahliae* showed rosette yellowing and premature senescence within 21 days (Fradin and Thomma, 2006; Reusche et al., 2014). Symptoms like tissue maceration occurred in *P. patens* within 24 h after *C. gloeosporioides* inoculation (Otero-Blanca et al., 2021), while the same concentration of conidia suspension droplets were added to the leaves of *A*. *thaliana*, the leaves wilted, chlorotic and exhibited water--soaked lesions after 3 days (Gao et al., 2021). Oomycetes *P. irregulare* and *P. debaryanum* were inoculated with *P. patens* by agar block, stem browning and tissue maceration was observed 1 day post inoculation, with increasing rotting and eventual death around 2 days as infection progressed (Toth and Birch, 2005; Oliver et al., 2009). However, after 2 days inoculation, *A*. *thaliana* with *P. irregulare* agar block, causing leaf wilting and brown, watery lesions symptoms (Castro et al., 2016). When *P. patens* is spray-inoculated with *P. carotovorum*, browning in stems and rhizoids can be observed within 1 day (Alvarez et al., 2016), whereas around 2 days are required to observe disease symptoms of water-soaked rotting leaves when *A. thaliana* was dipped in the bacterial suspension. The leaves of *P. patens* were soaked in the suspension of *P*. *syringae* at a concentration of 1 × 10^8^ cfu/mL. After 3 days, the leaves appeared bacterial spots, yellowing, wilting and other phenomena (Yan et al., 2023), while the higher concentration of bacteria was used to soak *A*. *thaliana*, the symptoms were observed after 4 days (Summermatter et al., 1995). Soft rot *E. carotovora* also displayed faster pathogenesis in *P. patens* compared to *A. thaliana* (Kariola et al., 2005; De León et al., 2007). The same is true for viruses. For example, Tomato spotted wilt virus (TSWV) inoculation of simultaneously in *P*. *patens* and *A*. *thaliana*, and the structural protein of the virus could be detected in *P*. *patens* after 11 days. In contrast, *A*. *thaliana* could be detected after 21 days, indicating that the incidence of TSWV in *P*. *patens* was faster than that in *A*. *thaliana* (Hühns et al., 2003).

**TABLE 2:** Comparison of susceptibility of moss and angiosperms to pathogens.

Pathogens	Hosts (Represented Moss/Angiosperm)	Germ inoculation method (Pathogen concentration)	Latent period of disease/d	Symptom	Reference
*Botrytis cinerea*	*Physcomitrium patens*	Spray spore suspension (5 × 10^6^ pieces/mL)	1	The leaves appeared brown spots, rotted and wilted	Yan et al. (2018)
	*Arabidopsis thaliana*	spore suspension (1,000 spores/μL)	1	Brown spots, yellowing, wilting, lodging, and rotting of leaves	González et al. (2006)
*Verticillium dahliae*	*Physcomitrium patens*	Spore suspension impregnation	15	Typical V-shaped necrotic spots appeared in leaves	(Unpublished data)
	*Arabidopsis thaliana*	Conidia suspension was incubated and dipping (1 × 10^6^ spores/mL)	21	Chlorosis of rosette leaves and symptoms of early senescence	Fradin and Thomma (2006), Reusche et al. (2014)
*Colletotrichum gloeosporioides*	*Physcomitrium patens*	Spray spore suspension (5 × 10^5^ pieces/mL)	1	Soaking and necrosis	Otero-Blanca et al. (2021)
	*Arabidopsis thaliana*	Drops of conidial suspension (5 × 10^5^ conidia/mL)	3	Fading green, wilting, water-soaked lesions	Gao et al. (2021)
*Pythium irregulare*	*Physcomitrium patens*	0.5 cm diameter agar block inoculation	1	The stem is brown and soaked soft	Oliver et al. (2009)
	*Arabidopsis thaliana*	0.5 cm diameter PDA plugs	2	Leaves chlorotic, rotten, watery spots	Castro et al. (2016)
*Pectobacterium carotovorum*	*Physcomitrium patens*	Spray	1	Browning of stems and roots	Alvarez et al. (2016)
	*Arabidopsis thaliana*	Dipping Conidia suspension (1 × 10^7^ cfu/mL)	2–3	Root rotting and water-soaked lesions on leaves	Sivaranjani et al. (2016)
*Pseudomonas syringae*	*Physcomitrium patens*	Soaking in bacterial Suspension (1 × 10^8^ cfu/mL)	3	Bacterial spots, browning, wilting, and yellowing of gametophytic leaves	Yan et al. (2023)
	*Arabidopsis thaliana*	Dipping Conidia suspension (4 × 10^8^ cells/mL)	4	Leaf chlorosis, yellowing	Summermatter et al. (1995)
*Erwinia carotovora*	*Physcomitrium patens*	Spray (5 × 10^8^ cfu/mL)	<2	Tissue damage with brown stems and decay	De León et al. (2007)
	*Arabidopsis thaliana*	Dropping after mechanical damage (5 × 10^5^ cfu/mL)	3	Tissue impregnation, drying	Kariola et al. (2005)
Tomato spotted wilt virus	*Physcomitrium patens*	Inoculation infection by rubbing plant leaves	11	The infection rate was low, no obvious symptoms	Hühns et al. (2003)
	*Arabidopsis thaliana*	Inoculation infection by rubbing plant leaves	21	Leaf yellowing and wilting	Hühns et al. (2003)

Current research reports indicate that several pathogens have been primarily isolated and identified from mosses, highlighting the unique host-pathogen interactions in bryophyte systems. Maybe these findings represent only a fraction of the potential moss-specific pathogens yet to be discovered. For example, after infecting *P. patens*, *M. racemosus* caused symptoms of tissue browning and leaf wilting around 3 days post inoculation, with gradual moss death starting after 1 week (Liu, 2011). *S. sphagnicola* is a potentially destructive necrotrophic pathogen capable of infecting *S. fuscum*. When *S. fuscum* tips were placed upside down on hyphae to inoculate the pathogen, yellowing was observed, and after 12–15 days, leaf necrosis occurred, causing the plants to become wrinkly and fragile (Tsuneda et al., 2001b). After artificially inoculating healthy *S. fuscum*, diseased plants showed brown lesions similar to those naturally infected. Detailed electron microscopic examination revealed the penetration of *S. fuscum* cell contents and walls by *S. sphagnicola* hyphae (Koukol and Kovarova, 2007). *A. muscivora* with *F*. *hygrometrica* as primary host can also infect *Hylocomium splendens*, *Aulacomnium palustre* and *Polytrichum juniperinum*, causing necrosis and wilting in all hosts (Davey et al., 2009). *Rozellopsidalean fungi* was only found in bryophytes, infecting *F. hygrometrica*, *B*. *pseudotriquetrum* and *B. capillare*, with browning observed at the penetration site and lateral shoots turning brown (Martínez-Abaigar et al., 2005). These findings collectively underscore the importance of studying moss-specific pathogens to better understand the unique aspects of plant-microbe interactions in non-vascular plants and their potential implications for broader plant pathology research.

## 4 Comparison of moss and vascular plant defense mechanisms against pathogens

### 4.1 Differences in physical defenses

Over a long period, plants and pathogens have evolved together. Plants utilize their cell walls as the first layer of defense against pathogenic microbial diseases, and modifications to these walls play a crucial role in their defense mechanisms (De León, 2011). Plant cell walls are composed of cellulose, hemicelluloses, pectin, xyloglucan, and hydroxyproline-rich proteins. In contrast, moss cell walls are thinner, lacking a clear distinction between primary and secondary walls. Additionally, mosses do not contain lignin but have polymers that are similar to lignans or lignin (lignans) (Ligrone et al., 2002; Popper, 2008). When pathogens invade, plants enhance their cell wall defense by incorporating phenolic compounds, depositing callose, and activating Dirigent (DIR) protein-coding genes involved in the synthesis of similar lignin compounds (De León et al., 2012; Reboledo et al., 2015; Alvarez et al., 2016). Moss can also release phenolic compounds from their thallus to prevent the germination of fungal spores and produce secondary metabolites to mitigate biological stress (Dixon, 2001; Commisso et al., 2021).

### 4.2 Differences in signaling regulation

Plants have evolved intricate signaling systems for perception, transduction, and response as a means of defending against pathogen invasion (Gabriel and Rolfe, 1990; Boller and Felix, 2009; Takken and Tameling, 2009). The pattern recognition receptors (PRRs) positioned on the plasma membrane of angiosperms are responsible for detecting conserved pathogen-associated molecular patterns (PAMPs) and inducing PAMP-triggered immunity (PTI), which offers protection against non-adapted pathogens (Jones and Dangl, 2006; Zipfel, 2009). Pathogens that are well-adapted to their host plants deliver effector molecules into plant cells to target key PTI components and suppress plant defenses (Boller and Felix, 2009; Boller and He, 2009). In response, plants possess a second layer of immune receptors encoded by resistance (R) genes. These receptors can detect effectors either directly or indirectly, leading to effector-triggered immunity (ETI). ETI is a highly specific immune response that is frequently accompanied by a hypersensitive response (HR) and systemic acquired resistance (SAR). Several PAMPs and their corresponding PRRs have been identified so far in angiosperms. In *A. thaliana*, the receptors FLS2 (Flagellin-Sensing 2), EFR1 (Elongation Factor Tu Receptor 1), and LYK1/CERK1 (LysM-containing Receptor-like Kinase1/chitin Elicitor Receptor Kinase1) recognize bacterial flagellin, Elongation Factor Tu, and fungal chitin, respectively (Gómez-Gómez and Boller, 2000; Zipfel et al., 2006; Miya et al., 2007), with LYK4 and LYK5 implicated as crucial for chitin signaling and immunity in *A. thaliana* (Wan et al., 2012; Cao et al., 2014). LYK5 has been identified as the chitin receptor in *A. thaliana*, forming chitin-induced receptor complexes with CERK1 to activate plant immunity (Cao et al., 2014). *P. patens* lacks close relatives of the FLS2 and EFR receptors (Boller and He, 2009), which aligns with findings that moss cells do not respond to flagellin (flg22) and Elongation Factor Tu (Bressendorff et al., 2016). A functional CERK1 receptor has recently been found in *P. patens* that can perceive PAMPs such as fungal chitin and bacterial peptidoglycan (Bressendorff et al., 2016). Mutation in *PpCERK1* led to reduced defenses, including lower expression of defense genes and reduced levels of cell wall-associated phenolic compounds, suggesting that PTI is an ancient plant defense response (Bressendorff et al., 2016). Further studies are needed to understand chitin perception in *P. patens*, including the analysis of molecular complexes associated with *PpCERK1* and a potential LYK5-like receptor, to better understand the immune response in moss.

Studies show that *P. patens* utilizes mechanisms for pathogen recognition that are similar to those found in vascular plants, as evidenced by the presence of typical R genes in its genome (De León, 2011; De León and Montesano, 2013). It also activates additional defense responses, such as the production of reactive oxygen species (ROS), programmed cell death (PCD), cell wall reinforcement, and increased expression of defense-related genes, which mirror the responses observed in angiosperms upon pathogen attack (De León et al., 2007; De León and Montesano, 2017). One of the initial plant responses following pathogen recognition is the production of ROS, which directly damages pathogens and signals cell wall strengthening, HR induction and gene expression changes (Torres et al., 2006). Conversely, necrotrophic pathogens appear to enhance the production of ROS by damaging host cells, leading to their death (Govrin and Levine, 2000). Typical hallmarks of PCD observed in pathogen-infected moss tissues include chloroplast degradation, accumulation of autofluorescent compounds, cytoplasmic shrinkage, nuclear fragmentation and nuclease activity (De León et al., 2007; De León et al., 2012; Wang et al., 2015). Several canonical intracellular receptor genes have been identified in *P. patens*, including kinase-Nucleotide Binding Site (NBS)-Leucine Rich Repeat (LRR) receptors and Toll/interleukin-1 like Receptor (TIR)-NBS-LRR (Akita and Valkonen, 2002; Xue et al., 2012; Tanigaki et al., 2014). Previous studies have shown that algae lack the homologous genes of NBS-LRR, TIR-NBS-LRR or TIR-LRR (Sarris et al., 2016), but recent studies have found that algae may have the original form of NBS-LRR or TIR-NBS-LRR genes (Andolfo et al., 2019; Andolfo et al., 2020). It is speculated that the origin and differentiation of RNL subclass are earlier than the separation of green algae and charophytes (Shao et al., 2019), and the emergence of NLR genes is related to the origin of terrestrial plants. This suggests that the evolution of receptor-like genes may represent an adaptive strategy for sensing pathogens and triggering defenses, which plays an important role in plant adaptation to terrestrial environments. The NBS-LRR protein family in terrestrial plants has expanded in vascular plants, which may be caused by polyploidization or paleopolyploidization events. The NBS-LRR protein family has expanded in vascular plants, likely due to polyploidy or ancient polyploidization events. Intriguingly, *Selaginella tamariscina* possesses a much smaller number of NBS-LRRs and other potential receptor genes compared to *P. patens* (Sarris et al., 2016). Further research is needed to explore whether pathogen effectors can inhibit moss defenses and if certain receptor-like proteins in activated *P. patens* can detect these effectors, either directly or indirectly, to initiate ETI.

In angiosperm, calcium ions (Ca^2+^) play a vital role as a second messenger in PTI and ETI responses, in the process of plant-microorganism interaction, the change of intracellular Ca^2+^concentration is one of the earliest biochemical characteristics detected after microbial recognition. leading to the production of ROS and (nitric oxide) NO, as well as the activation of defense gene expression (Seybold et al., 2014; Zhang et al., 2014). The signaling of biotic stress in plants is regulated by calcium-dependent protein kinases (CDPKs) and mitogen-activated protein kinase (MAPK) pathways, both of which elevate cytoplasmic Ca^2+^ levels upon perception of pathogens (Seybold et al., 2014). In *P. patens*, chitin oligosaccharide treatment resulted in Ca^2+^ oscillations, and calcium carriers could stimulate expression of defense-related genes. The influx of Ca^2+^ alone was enough to provoke defense responses similar to those observed in angiosperms (Galotto et al., 2020).

### 4.3 Conservation and evolutionary specificity of immune components in mosses

**FIGURE 1:**
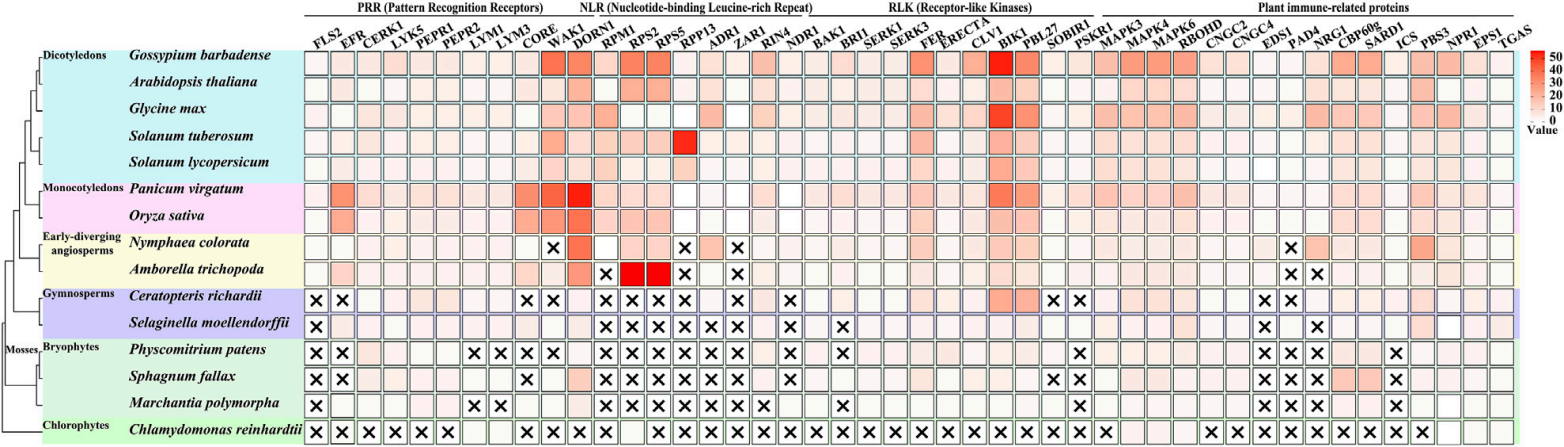
Evolutionary distribution of immune-related genes across plant lineages. This figure illustrates the presence and copy number of key immune-related genes across 15 representative plant species, spanning from chlorophytes to angiosperms, with a focus on mosses. The heatmap represents the number of orthologous genes for each immune component (columns) in each species (rows), with darker colors indicating higher copy numbers. Data acquisition and analysis: Genome sequences were obtained from Phytozome (https://phytozome-next.jgi.doe.gov/). The following genome versions were used: *Chlamydomonas reinhardtii* CC-4532 v6.1, *Marchantia polymorpha* v3.1, *Sphagnum fallax* v1.1, *Physcomitrium patens* v6.1, *Selaginella moellendorffii* v1.0, *Ceratopteris richardii* v2.1, *Amborella trichopoda* v1.0, *Nymphaea colorata* v1.2, *Oryza sativa Kitaake* v3.1, *Panicum virgatum* v5.1, *Solanum lycopersicum* ITAG5.0, *Solanum tuberosum* v6.1, *Glycine max* Wm82. a6. v1, *Arabidopsis thaliana* Araport11, and *Gossypium barbadense* v1.1. Orthologous genes were identified using OrthoFinder (Emms and Kelly, 2019).

A comprehensive analysis of immune-related gene families across plant lineages has revealed intriguing patterns of conservation and innovation (Figure 1), particularly in mosses. The examination of key immune components, including PRRs, Nucleotide-binding Leucine-rich Repeat (NLR) proteins, Receptor-like Kinases (RLKs), and other plant immune-related proteins across various plant groups (Wu and Zhou, 2013), has uncovered significant insights into the evolution of plant immunity.

In the realm of Pattern Recognition Receptors, a clear evolutionary trend in their diversification is observed. Notably, the bacterial PAMP receptors FLS2 and EFR are absent in chlorophytes and mosses, only emerging in lycophytes and becoming more diverse in angiosperms (Wu and Zhou, 2013; de Vries et al., 2018; Delaux and Schornack, 2021). This suggests that these specific bacterial recognition mechanisms evolved after the divergence of mosses from the plant lineage leading to vascular plants. In contrast, mosses possess multiple copies of the chitin-sensing receptors CERK1 and LYK5, with numbers comparable to or exceeding those found in many angiosperms. This indicates an early evolution and potential importance of fungal pathogen recognition in mosses. The presence of PEPR1 and PEPR2, receptors for damage-associated molecular patterns (DAMPs), in mosses but with lower copy numbers compared to vascular plants, suggests a more ancient origin of DAMP recognition systems (Engelsdorf et al., 2018; Liu et al., 2024). One of the most striking observations is the complete absence of canonical NLR genes (such as RPM1, RPS2, RPS4, RPS5, RPP13) in mosses and other non-vascular plants. This finding suggests that the complex NLR-mediated immunity, crucial for effector-triggered immunity, evolved after the divergence of vascular plants, representing a major innovation in plant immune systems. This absence implies that mosses must rely on alternative strategies for pathogen resistance. The analysis of RLKs revealed a mixed pattern of conservation and innovation. Co-receptors such as BAK1, SERK1, and SERK3 are present in mosses, indicating an early evolution of these signaling components (Ngou et al., 2024).

The examination of other immune-related proteins yielded several noteworthy findings. The MAPK cascade components (MAPK3, MAPK4, MAPK6) are present in similar numbers in mosses and vascular plants, suggesting early evolution and conservation of this signaling module (Ghelis, 2011; Ramírez-Zavaleta et al., 2022; Singh et al., 2024). RBOHD, involved in ROS production, is also present in mosses, indicating an ancient origin of this defense mechanism (Marchetti et al., 2024). However, key regulators of basal immunity and systemic acquired resistance, EDS1 and PAD4, are absent in mosses, correlating with the lack of canonical NLRs (Ramírez-Zavaleta et al., 2022). These observations collectively paint a picture of moss immunity as a unique system that combines ancient, conserved elements with lineage-specific innovations. The absence of certain immune components (e.g., FLS2, EFR, NLRs) coupled with the expansion of others (e.g., CERK1, CBP60g) suggests that mosses have evolved alternative strategies to cope with pathogens. The expansion of chitin recognition receptors implies an enhanced capacity for fungal pathogen detection, possibly reflecting the importance of fungal interactions in their evolutionary history. The absence of canonical NLRs suggests a heavier reliance on PTI rather than ETI for pathogen resistance in mosses. Furthermore, the expansion of salicylic acid-related transcription factors could indicate a novel regulatory mechanism for defense responses, potentially compensating for the lack of NLR-mediated immunity.

In conclusion, the unique immune profile of mosses, characterized by both the absence of seemingly crucial components and the expansion of others, challenges conventional understanding of plant immunity. It suggests that there may be alternative, yet undiscovered mechanisms of pathogen resistance in these ancient plant lineages, which could potentially inform new strategies for crop protection and disease resistance in the future.

### 4.4 Differences in gene regulation

In angiosperms, AP2/ERFs (APETALA2/Ethylene Responsive Factors) play important regulatory roles in plant defenses against pathogens and abiotic stresses by controlling the expression of their target genes (Xu et al., 2011). On the other hand, a number of ERF family members are induced in *P. patens* during pathogen infection, indicating their role as immune modulators in mosses (Reboledo et al., 2022a). Researchers studied a pathogen-induced ERF called PpERF24 in *P. patens* and found that its direct orthologs exist only in other mosses, being absent in the bryophytes *Marchantia polymorpha* and *Anthoceros agrestis*, the vascular plant *S. tamariscina,* or angiosperms (Reboledo et al., 2022b). This demonstrates that PpERF24 belongs to a moss-specific clade with unique amino acid features in the AP2 DNA-binding domain. Interestingly, all members of the PpERF24 sub-clade are induced by fungal pathogens, making PpERF24 a unique pathogen-responsive gene in moss (Reboledo et al., 2022b). Among the upregulated differentially expressed genes during *B. cinerea* infection, 216 were specifically expressed in *P. patens*, whereas showing minimal or no expression in other vascular plants (Reboledo et al., 2021). Additionally, 22 genes encoding putative fungal major facilitator superfamily transporters were also upregulated in *P. patens*, but typically exhibited no expression or reduced expression in vascular plants (Reboledo et al., 2021). Other specifically upregulated genes in *P. patens* included carboxylic ester hydrolases, cellobiose dehydrogenase, β-glucosidases, peroxidase, polyketide synthases and numerous hypothetical proteins. Additionally, *Bcaba2*, which encodes a cytochrome P450 monooxygenase involved in abscisic acid (ABA) biosynthesis, was only upregulated in *P. patens* tissues after a sustained 24-h infection and was absent in vascular plants (Reboledo et al., 2021).

The activation of defense responses upon pathogen infection involves the induction of various host genes (Wise et al., 2007). Some pathogen-induced genes encode enzymes involved in synthesizing antimicrobial compounds, oxidant stress protection enzymes, tissue repair enzymes, and cell wall fortification enzymes, while others encode proteins with regulatory functions in defense signaling pathways. When *B. cinerea* infects *P. patens*, there is an increased expression of phenylalanine ammonia lyase (PAL), chalcone synthase (CHS), lipoxygenase (LOX) and the classic vascular plant host defense marker pathogenesis-related protein PR-1 (Oliver et al., 2009; De León and Montesano, 2013). The enzymes encoded by these genes contribute to the synthesis of phenylpropanoids, flavonoids, and oxygenated fatty acids, which play various roles in defense responses (Feussner and Wasternack, 2002; De León and Montesano, 2013). Additionally, genes associated with PCD are also upregulated, encoding proteases, nucleases and Bax Inhibitor-1 proteins that regulate PCD (De León and Montesano, 2013). The genome of *P. patens* contains many gene family members involved in phenylpropanoid metabolism (Rensing et al., 2008). *P. patens* has a higher number of members composing PAL and CHS multigene family members as compared to vascular plants (Koduri et al., 2010; Wolf et al., 2010), and some of these are induced upon pathogen attack (De León et al., 2007; Oliver et al., 2009). These enzymes can produce novel metabolites that may function in impeding pathogen infection. Moreover, the presence of oxylipids (produced by LOX) in *P. patens*, which are not found in vascular plants, makes it a valuable model for identifying new defense related compounds and defense mechanisms that may have evolved or been lost in plants over time (Oliver et al., 2009).

### 4.5 Differences in hormone regulation

Plants resist pathogenic microbes by altering hormone levels and expression of defense genes. Salicylic acid (SA), jasmonic acid (JA), and ethylene (ET) play important roles in host responses to pathogen infections (Glazebrook, 2005; Lorenzo and Solano, 2005; Van Loon et al., 2006; Loake and Grant, 2007; Oliver et al., 2009). SA is primarily associated with resistance against biotrophic pathogens and serves as an important endogenous signal for triggering HR and SAR (Saleem et al., 2021). In contrast, JA and ET are typically involved in responses to necrotrophic pathogens, with their signaling pathways working synergistically to produce induced systemic resistance (ISR) (Jiang et al., 2019). These hormones also influence susceptible responses. Generally, the SA and JA pathways are antagonistic, while the JA and ET pathways are synergistic (Xie and Duan, 2023). JA also confers resistance against (hemi)biotrophic pathogens in rice (De Vleesschauwer et al., 2014). And studies have shown that JA activation while abscisic acid signal inhibition may be a key factor in activating the basic response to fungal resistance (Amoroso et al., 2023). Additionally, plant hormones such as abscisic acid and auxin are also involved in PAMP-triggered immune responses. Upon pathogen invasion, JA-isoleucine rapidly accumulates in angiosperms, promoting the interaction between the receptor COI1 (Coronatine-insensitive protein 1) and inhibitory protein JAZ (Jasmonate-ZIM domain) (Chini et al., 2007; Jeong et al., 2023). Although *P. patens* encodes orthologs of all JA signaling components, the role of JA in moss disease resistance remains unclear (De León et al., 2015). Instead, its precursor cis-oxophytodienoic acid (OPDA) accumulates in moss tissues after pathogen attack (Oliver et al., 2009; De León et al., 2012). Both methyl jasmonate and OPDA can induce the expression of the defense gene *PAL*. Similar to *A. thaliana*, OPDA can act as a signaling molecule in mosses, resulting in the induction of defense-related genes (Taki et al., 2005). In mosses, SA treatment also induces expression of *PAL*. ABA contributes to the synthesis of papillae upon pathogen perception and also promotes the expression of certain defense genes (Adie et al., 2007). In *P. patens*, ABA induces the synthesis of defense proteins, including the RPM1-related R protein, LOX, an intracellular pathogenesis-related protein, a N-hydroxycinnamoyl/benzoyl transferase involved in phytoalexin production, a hydroxyproline-rich glycoprotein, and a proline-rich protein that aids in cell wall reinforcement, as well as ascorbate peroxidase and peroxiredoxin, indicating ABA may play a role in moss defenses against pathogens (Wang et al., 2010; De León and Montesano, 2017). While auxin can promote wound healing tissue formation in plants, it has a negative regulatory effect during biotrophic pathogen infections. Auxin plays significant roles in resistance against *B. cinerea* and *P. carotovorum* (Llorente et al., 2008). It is also implicated in moss defenses, with auxin signaling responding to infection by *P. irregulare*, *P. debaryanum*, and *C. gloeosporioides* (Mittag et al., 2015; Reboledo et al., 2015; Alvarez et al., 2016), and can induce *PAL* expression (Reboledo et al., 2015). The roles of cytokinin and brassinosteroid in moss defenses have yet to be reported. Overall, these phytohormone signaling pathways have played critical roles in the adaptation of land plants to microbial pathogens (De León and Montesano, 2017).

## 5 Gene mining and application of mosses

Gene mining in mosses can provide promising insights for stress-resistant breeding. Currently, a number of disease-resistant genes have been identified in mosses and successfully utilized in vascular plants. *Soloist* gene, which encodes a unique subfamily member of the AP2/ERF transcription factor family, plays an important role in plant responses to both biotic and abiotic stresses. Overexpression the *Soloist* gene (*ScAPD1-like* gene) found in *S. caninervis* significantly increased the ability of transgenic *A. thaliana* and *S. caninervis* to combat *V. dahliae,* reduced ROS accumulation, and improved their scavenging ability (Li et al., 2023). Additionally, the ectopic expression of *PpBURP2* from *P. patens* in rice enhanced tolerance to osmotic and salt stresses, as well as drought stress. Overexpressing *PpBURP2* in rice can enhance resistance to bacterial leaf blight (Yu et al., 2022). Cinnamyl alcohol dehydrogenase (CAD) is a key enzyme in the final step of lignin biosynthesis. *PpCAD1* can inhibit the invasion of *B. cinerea* by boosting phenylpropanoid levels in the cell wall. Studies have shown that plants overexpressing *PpCAD1* in *P. patens* exhibit enhanced resistance to *B. cinerea*, while knockout mutants show reduced resistance (Jiang et al., 2022). In the vascular plant *A*. *thaliana*, *AtCAD1* is also essential for lignification (Eudes et al., 2006), and the *AtCAD1* gene negatively regulates the cell death aspect of the plant immune response by SA signaling pathway (Morita-Yamamuro et al., 2005). However, ectopic expression of *PpCAD1* in *A. thaliana* significantly enhanced its tolerance to *B. cinerea* (Jiang et al., 2022). In addition, ectopic expression of the pathogenesis-related (PR) −10 gene (*PpPR-10*) from *P*. *patens* could enhance the resistance of *A. thaliana* to *P*. *irregulare*, which was proved by smaller lesions and less cell damage compared with wild-type plants (Castro et al., 2016). These findings suggest that the discovery of moss genes can be effectively applied to other vascular plants, indicating that studying moss-pathogen interaction can inspire new strategies for molecular breeding aimed at enhancing plant disease resistance.

## 6 Summary and prospect

The study of plant pathology has traditionally centered on the interaction between pathogens and plants. Recent findings indicate that fungi, bacteria, and viruses can also infect mosses. Due to their small size, simple structure, and short life cycles, mosses present an excellent model systems for studying plant-pathogen interactions. Mosses are more suitable for genetic manipulation, allowing for extensive gene deletion and overexpression studies compared to vascular plants (Zhou et al., 2024). Despite the fact that vascular plants and mosses share similar pathogen sensing mechanisms and downstream pathways, many issues in this field remain unresolved. Moreover, mosses like *P*. *patens* also have a telomere-to-telomere (T2T) genome (Bi et al., 2024) and extensive data from multiple histological studies, making them ideal for in-depth research on plant-pathogen interactions. Although there have been numerous recent publications on moss-pathogen interactions, further investigation is necessary to fully understand the underlying molecular mechanisms. Firstly, future research could focus on expanding the scope of studies to interactions between different moss species and significant pathogens. As mentioned above, current knowledge of moss-pathogen interactions is limited, primarily concentrating on a few model mosses and typical pathogens. Different moss species may exhibit varying responses and interacting mechanisms against the same pathogen. Therefore, more systematic studies are needed to explore the interactions between diverse moss species and important pathogens, which is crucial for understanding disease resistance mechanisms in mosses.

**FIGURE 2:**
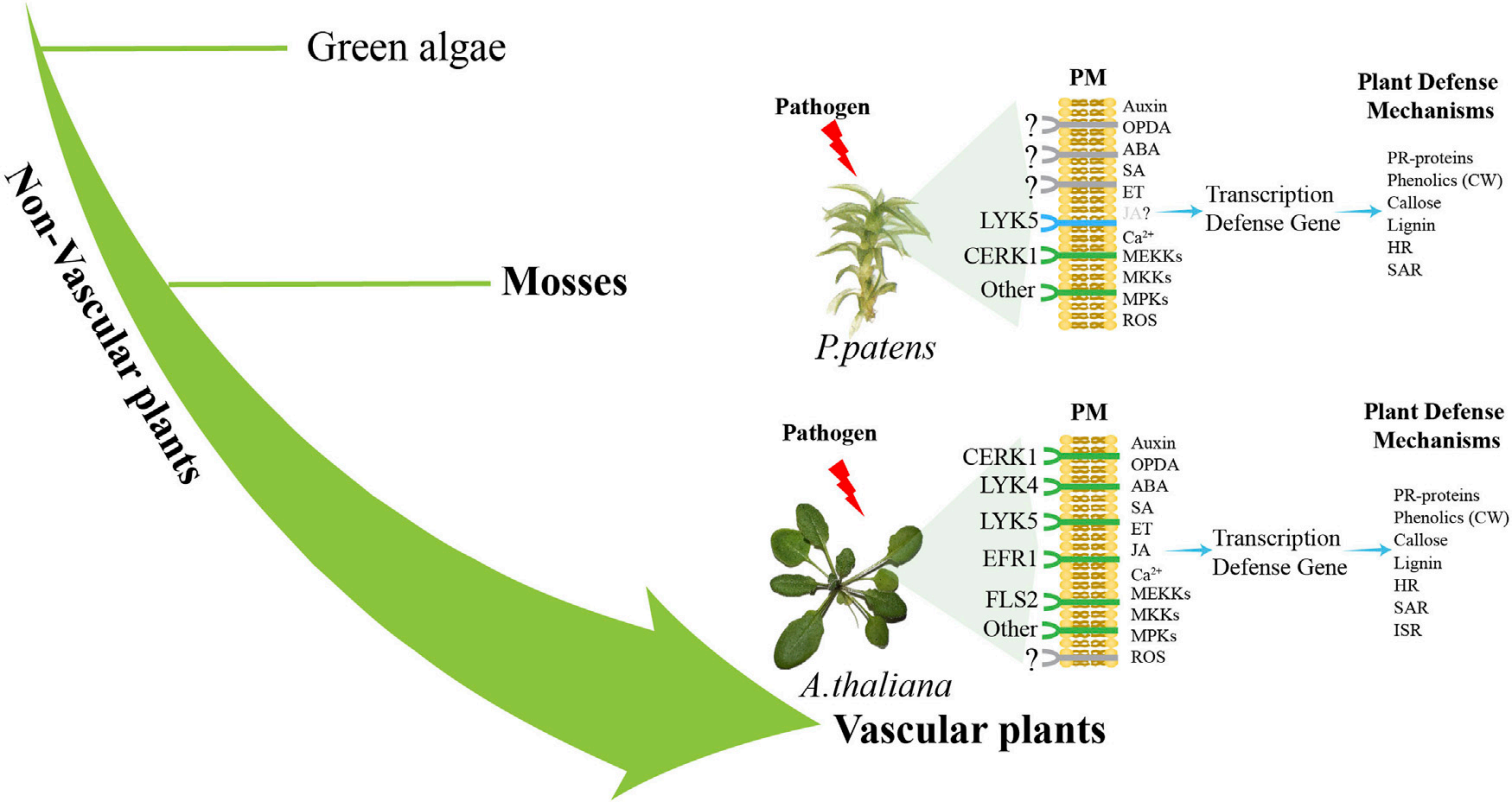
Difference pathways of pathogen sensing between vascular plants and non-vascular plants. Plants sense pathogen-associated molecular patterns (PAMPs) such as FLS2, EFR1, CERK1, and LYK5 through plasma membrane (PM) PRRs (Couto and Zipfel, 2016). Pathogen recognition triggers Ca^2+^production and activates the MAPK cascade (Seybold et al., 2014). *P. patens* lacks the close relatives of receptors FLS2 and EFR, while a functional CERK1 receptor senses fungal chitin and bacterial peptidoglycan (Bressendorff et al., 2016). Subsequently, at least one MAP kinase kinase (MEKKs), one MAP kinase (MKKs) and two MAP kinases (MPKs) are activated to participate in the defense response of moss to fungal chitin (Bressendorff et al., 2016). ROS, SA and auxin activate the expression of defense genes, leading to the activation of defense mechanisms (De León et al., 2012; De León and Montesano, 2013), including the expression of genes encoding PR proteins, the entry of phenols into cell wall (CW), callose deposition and the accumulation of pre-lignin compounds (Overdijk et al., 2016; De León and Montesano, 2017). HR-like reaction and SAR were also activated in infected mosses (De León et al., 2012). *A. thaliana* and other angiosperms have receptors such as LYK5, LYK4, CERK1, EFR1 and FLS2 (Couto and Zipfel, 2016), which activate MEKK, MKK and MPKs, leading to the production of ROS and the expression of defense genes (Meng and Zhang, 2013). Hormones SA, JA and OPDA, ABA, auxin and ET activate the expression of defense genes, leading to the activation of defense mechanisms, including PR proteins, phenolic substances into the CW, callose and lignin deposition, HR, SAR and ISR activation (Glazebrook, 2005). The green color is the identified immune-related receptor, and the blue color is the homologous gene of the receptor identified by genomic comparison.

Secondly, improving the investigations of the molecular pathways that govern how mosses detect pathogens and trigger immune response is essential. Our current understanding of the morphological, physiological, invasion strategies, sensing mechanisms, and downstream signaling pathways in moss-pathogen interactions is quite limited. According to the existing reports, it is found that *P*. *patens* lack many receptors that already exist in *A. thaliana*, such as CREK1 and LYK5, while retaining some basic modules of the immune system (Figure 2), which reveals that bryophytes may adopt different immune strategies from angiosperms. At present, the research on moss immune pathway is still relatively scarce. Subsequent investigations may employ multi-omics and bioinformatic approaches to comprehensively analyze regulatory changes at various levels in pathogen-infected mosses, thereby identifying key immune-related genes and signals associated with immunity. To gain deeper insights into the specificity of immune regulation in plants and the conservation of evolutionary traits, it is important to enhance comparisons between mosses and vascular plants.

Thirdly, creating a high-throughput screening platform based on moss mutants is also recommended. Mosses are ideal for high-throughput gene knockout or overexpression due to their small size, short cultivation cycle, and high frequency of homologous recombination. These characteristics allow for large-scale mutant screening under pathogen infections, facilitating the identification of important genes or mechanisms that influence disease resistance.

Additionally, developing a rapid gene screening system in mosses for mining disease resistance genes is crucial. Mosses can serve as ideal platforms for validating plant resistance genes because they are easy to cultivate and support rapid pathogen growth. This allows for the efficient heterologous expression of both known and unknown resistance genes from vascular plants, enabling quick functional verification and high-quality gene selection, which can yield promising candidates for molecular plant breeding. In addition, with advancements in gene editing technology and crop genetic transformation systems (Lu et al., 2020; Cao et al., 2023; Tian et al., 2024), we can look forward to exciting breakthroughs in gene applications that will drive progress and innovation in agriculture and related fields.

The advancement of high-throughput omics technologies opens up opportunities for future research to conduct in-depth analyses of important activated genes, signals, and metabolites in pathogen-infected mosses. By combining gene editing and transgenic techniques to eliminate or overexpress key immune genes in mosses, researchers can gain a better understanding of their specific roles in plant immune responses. Furthermore, comparing how mosses react to various pathogens may reveal conserved immune mechanisms. Mosses are also excellent models for validating plant resistance genes. As our knowledge in this field expands, mosses will play a crucial role in elucidating the evolution of immune mechanisms in plants and will provide the theoretical foundations and technological support for developing disease-resistant crops.
